# Development and application of loop-mediated isothermal amplification (LAMP) for detection of *Plasmopara viticola*

**DOI:** 10.1038/srep28935

**Published:** 2016-07-01

**Authors:** Xiangjiu Kong, Wentao Qin, Xiaoqing Huang, Fanfang Kong, Cor D. Schoen, Jie Feng, Zhongyue Wang, Hao Zhang

**Affiliations:** 1 State Key Laboratory for Biology of Plant Diseases and Insect Pests, Institute of Plant Protection, Chinese Academy of Agriculture Sciences, Beijing, China; 2 Plant Research International, P.O. Box 69, 6700 AB, The Netherlands

## Abstract

A rapid LAMP (loop-mediated isothermal amplification) detection method was developed on the basis of the ITS sequence of *P. viticola*, the major causal agent of grape downy mildew. Among the 38 fungal and oomycete species tested, DNA isolated exclusively from *P. viticola* resulted in a specific product after LAMP amplification. This assay had high sensitivity and was able to detect the presence of less than 33 fg of genomic DNA per 25-μL reaction within 30 min. The infected leaves may produce sporangia that serve as a secondary inoculum. The developed LAMP assay is efficient for estimating the latent infection of grape leaves by *P. viticola*. When combined with the rapid and simple DNA extraction method, this assay’s total detection time is shortened to approximately one hour; therefore it is suitable for on-site detection of latent infection in the field. The sporangia levels in the air are strongly associated with disease severity. The LAMP method was also demonstrated to be able to estimate the level of sporangia released in the air in a certain period. This assay should make disease forecasting more accurate and rapid and should be helpful in decision-making regarding the control of grape downy mildew.

Grape downy mildew, caused by *Plasmopara viticola*, is one of the most important diseases of grapes worldwide. During the growing season, the pathogen can infect all green parts of the vine whenever the weather is warm and wet. After years of extremely favorable environmental conditions, a yield reduction as much as 80% may be observed in vineyards if control procedures are not implemented^1^. Currently, the disease has caused a large bottleneck restricting the development of the grape industry^2^,^3^.

At present, chemical control is the most effective remedy for grape downy mildew. Vine growers usually prevent the disease by spraying fungicide any time they foresee favorable meteorological conditions for pathogen outbreak. Forecasting models have contributed to the abandonment of calendar-based spraying. However, these simulators often alert growers to treat vines in the absence of infection, thus leading to the overuse of chemicals, environment pollution and a decrease in grape quality. Rapid and sensitive detection techniques for monitoring the presence and severity of infection would help growers to accept or disregard the alert of forecasting models and to apply chemicals with more accuracy and at a higher efficiency, without the risk of crop loss. Therefore, a rapid and low-cost detection method would be of great significance to the scientific management of grape downy mildew.

Loop-mediated isothermal amplification (LAMP) is a novel molecular biological detection technology, that specifically detects genomic DNA by using a set of six oligonucleotide primers specific to different regions of a target gene. This method has been widely applied in many fields for on-site detection because of its low cost, high specificity, efficiency, simplicity of operation, rapidness, and ability to be used in broad applications, such as disease diagnosis and food safety testing^4^.

Several applications of LAMP for the detection of fungal pathogens have been described to date. The detection of *Fusarium graminearum* has been described by Niessen and Vogela^5^. Tomlinson *et al*.^6^ have published a LAMP-based assay for the detection of the oomycete *Phytophthora ramorum*, which can cause sudden oak death disease. Most recently, Lu *et al*.^7^ have established a LAMP assay for the detection and identification of *Fusarium oxysporum* from soybeans. However, thus far, no attempt has been made to use LAMP for the detection of *P. viticola*.

In this study, we describe the development and application of a LAMP-based assay for the specific detection of *P. viticola* in infected leaves and the airborne sporangia collected from a spore trap. The use of the visual colorimetric indicator hydroxy naphthol blue for the in-tube detection of DNA amplification as well as simplified methods for the preparation of amplifiable target DNA is demonstrated and may widely improve disease management in the grape industry by allowing the pathogen to be monitored and making analyses easier and more cost effective.

## Results

### Design of primers and the LAMP assay

**Figure 1:**
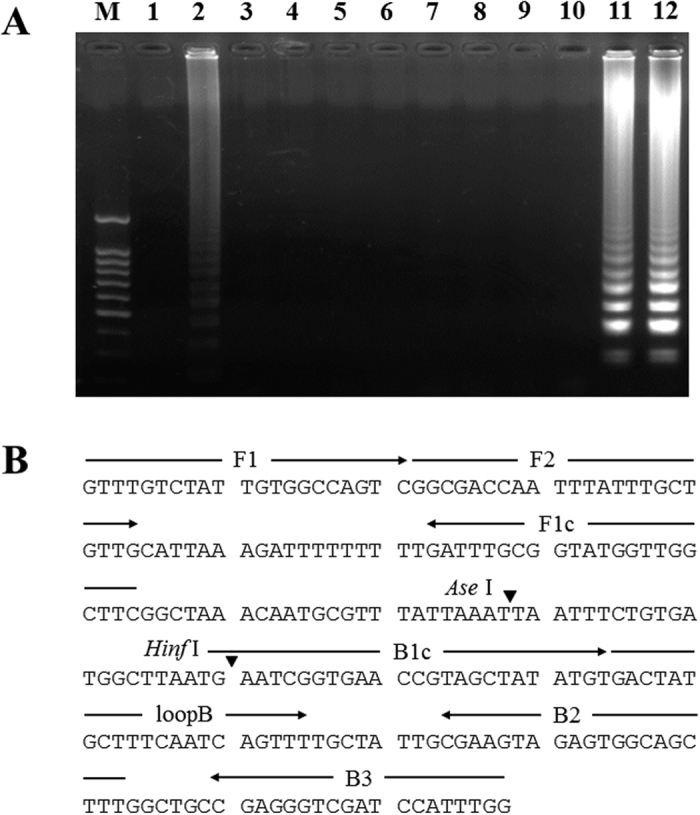
Optimization of LAMP primers. (**A**) The assessment of primer sets was based on gel electrophoresis analysis of the LAMP products. (**B**) The position and orientation of the selected LAMP primers within the nucleotide sequence of the ITS region of *P. viticola* (GenBank accession no. DQ665668.1).

The *P. viticola* ITS sequence (DQ665668.1) was chosen as the target region for the LAMP primers. Its full length is 2,337 bp, and it has an overall GC content of 43.3%. The 565-bp fragment (position 694 to 1258) of *P. viticola* was selected as the target sequence for LAMP primer design. Primer design was performed by using the online tool Primer Explorer version 4.0 (http://primerexplorer.jp/elamp4.0.0/index.html). All of the parameters were set by default. Twelve primer sets were screened, and two of them showed typical ladder-like DNA fragments on an agarose gel (Fig. 1A). For primer set 12, a loop primer was developed to accelerate the LAMP reaction. The positions of the primers within the ITS sequence are shown in Fig. 1B. On the basis of the alignment of the *cox2* gene of *Plasmopara viticola* (DQ365760), *Plasmopara halstedii* (EU743813), *Plasmopara obducens* (DQ365757), *Peronospora belbahrii* (FJ394342) and *Peronospora elsholtziae* (FJ527435), a specific primer pair for *P. viticola* was developed; the predicted size of the PCR product was 591 bp. The sequence of the LAMP and conventional PCR primers are listed in Table 1.

### Validation of the LAMP products

**Figure 2:**
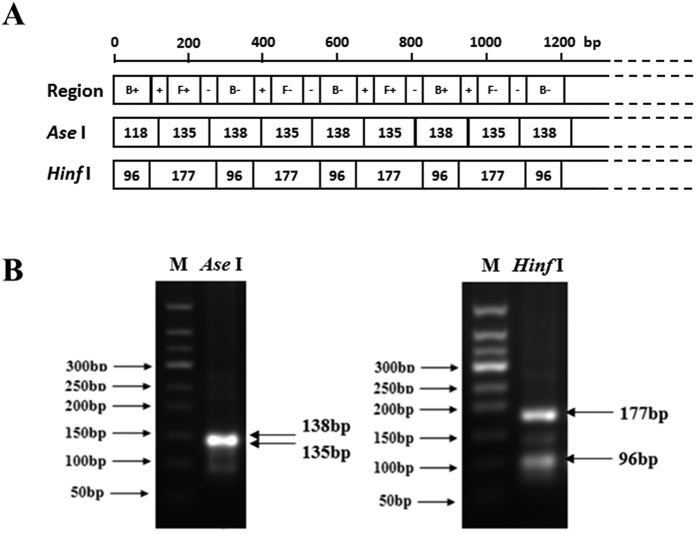
Restriction enzyme digestion of the positive LAMP products. (**A**) Schematic representation of the anticipated restricted DNA products. B+, B−, F+, F−,+ and-in the first row represent the same regions described by Notomi (Notomi *et al*.^4^). (**B**) LAMP products were digested with *Ase* I and *Hinf* I, and the fragments were observed by 2.0% agarose gel electrophoresis.

To validate the expected amplification products, LAMP products were digested with the restriction enzyme *Hinf* I or *Ase* I. The restriction sites for *Hinf* I and *Ase* I, as well as the sizes of the restriction fragments, are shown in Fig. 2A. After *Ase* I digestion, only one band was observed because of the similar sizes of the two fragments (135 bp and 138 bp). For *Hinf* I digestion, the anticipated 177 bp and 96 bp bands were clearly observed on an agarose gel (Fig. 2B). To further confirm the specificity of the LAMP products, the plasmid pMD228 was sequenced, and the results showed that the 228-bp target fragment was 100% homologous to the ITS sequence used for the primer design. These results indicated that the LAMP products were specifically amplified from the ITS target region in *P. viticola*.

### Specificity of the LAMP assay for *P. viticola*

**Figure 3:**
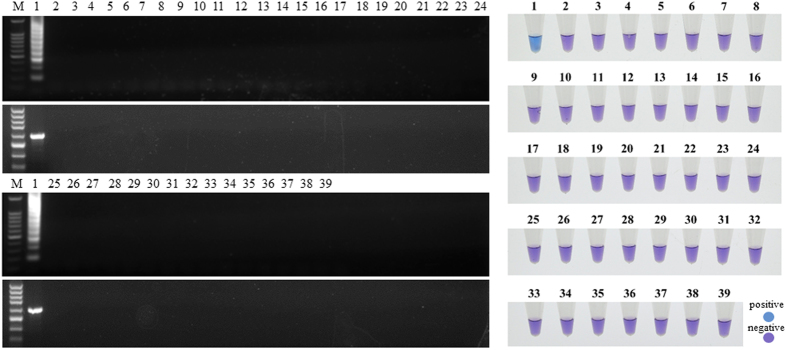
Specificity of LAMP detection vs. conventional PCR. The number of isolates is identical to that in Table 2, and No. 39 is the negative control.

The specificity of the LAMP primer set for the detection of *P. viticola* was analyzed by using DNA from the 38 strains listed in Table 2. The strains tested belonged to 25 genera, including 8 species within *Peronosporales* and 9 common grape pathogens. A positive sample was indicated by a sky-blue color and a ladder-like pattern on an agarose gel, whereas a negative sample remained a violet color. The sky-blue color and ladder-like pattern of bands were generated solely with the DNA of *P. viticola*. Neither species within *Plasmopara* nor those from other genera showed positive results under the same conditions (Fig. 3). This result indicated that the primer set can be used to specifically detect *P. viticola*. Conventional PCR with specific primers (Pv-cox2 F/R) also showed good specificity, and the target fragment was amplified with only the DNA of *P. viticola*.

### Sensitivity of LAMP assay for *P. viticola*

**Figure 4:**
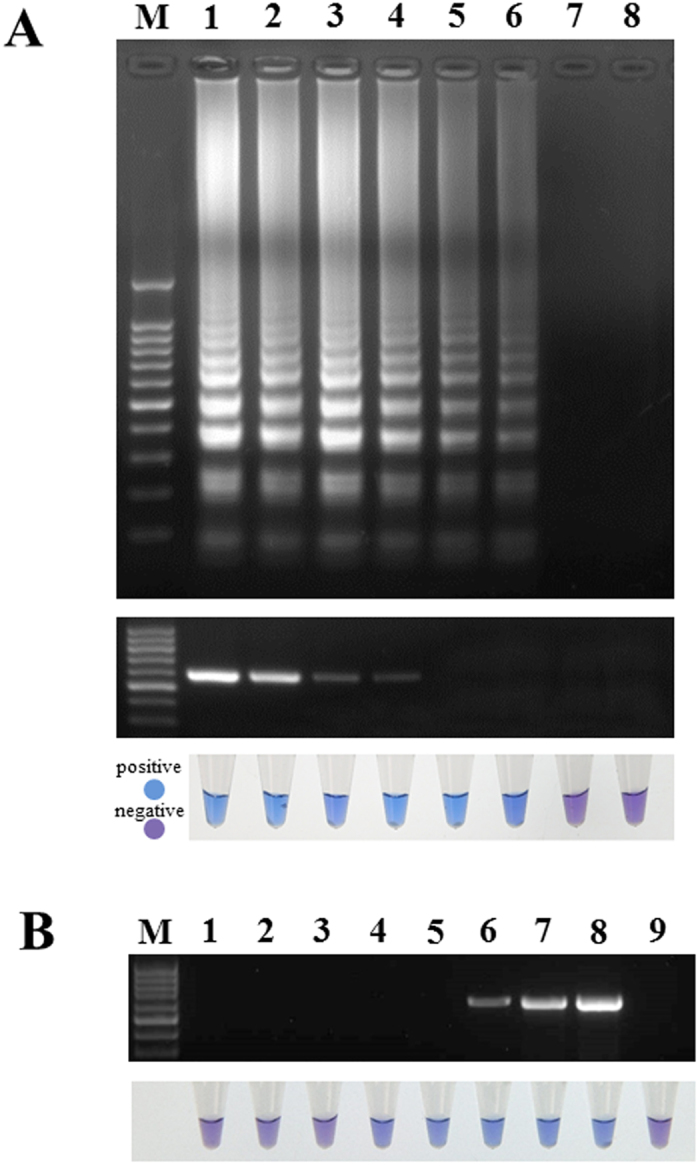
Sensitivity of LAMP vs. conventional PCR. (**A**) Sensitivity on purified DNA of *P. viticola*. M, 100-bp DNA Ladder; 1, 3.3 ng; 2, 330 pg; 3, 33 pg; 4, 3.3 pg; 5, 330 fg; 6, 33 fg; 7, 3.3 fg; 8, negative control. (**B**) Sensitivity on artificially infected grape leaves. M, 100-bp DNA ladder; 1, 1 dpi; 2, 2 dpi; 3, 3 dpi; 4, 4 dpi; 5, 5 dpi; 6, 6 dpi; 7, 7 dpi; 8, 8 dpi; 9, negative control.

Ten-fold serial dilutions of purified genomic DNA were used to evaluate the sensitivity of the method. The ladder-like pattern of bands and sky-blue color were observed from 3.3 ng to 33 fg per reaction, indicating that the detection limit was 33 fg of genomic DNA. For conventional PCR, a weak 591-bp fragment was amplified when the template DNA was diluted to 3.3 pg (Fig. 4). Thus, for purified DNA, LAMP was at least 100 fold more sensitive than conventional PCR.

Sensitivity was also tested on artificially inoculated grape leaves. The pathogen could be detected at 4 dpi using the LAMP assay. However, for conventional PCR, positive results were not observed until 6 dpi. These findings indicated that our LAMP assay is able to detect latent infection 2 days earlier than conventional PCR under the culture conditions in a laboratory setting.

### Application of LAMP on *P. viticola*-infected grape leaves

**Figure 5:**
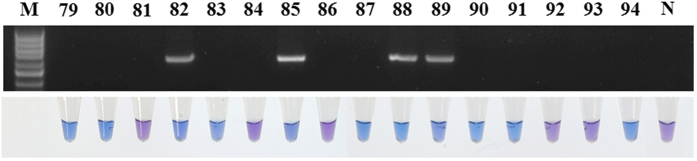
Application of LAMP and conventional PCR on grape leaves without visible symptoms. The number of samples is identical to that in Table S1. N; negative control.

LAMP and conventional PCR were performed on 150 grape leaf samples including the main varieties and grape-producing areas in China. LAMP and PCR were both positive for samples No. 1~78, on which white mildew was observed (Table S1). However, for the leaves without visible symptoms (No. 79~150), LAMP (47/72, 65.2%) scored significantly more samples positive compared with conventional PCR (16/72, 22.2%) (Fig. 5, Table S1), thus indicating that LAMP is more sensitive than conventional PCR for the detection of the latent infection of *P. viticola* in grape leaves. All of the non-infected samples tested showed negative results.

### Application of LAMP to monitor airborne sporangia in the field

**Figure 6:**
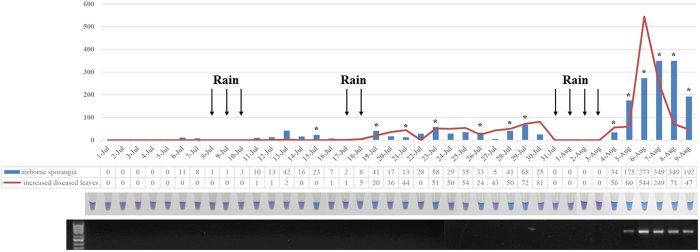
Application of LAMP in the monitoring of airborne sporangia in the field. Asterisks on the different columns represent the positive results of LAMP. The first tube and corresponding region in the agarose gel represent the negative control.

We investigated both the daily airborne sporangia and the increased numbers of diseased leaves for 40 consecutive days. *P. viticola* sporangia were first detected on July 6, and the number increased slowly until August 5, when a sharp increase was observed after the third rainfall period. The number of diseased leaves, monitored daily, showed a similar trend (Fig. 6). The LAMP assay was performed on all of the air samples. When the amount of sporangia was less than 20 (14 samples), only one sample was positive (7.1%). When the number was between 20 and 100, more positive samples were scored (66.7%; 8/12). All of the samples with more than 100 sporangia were positive. For conventional PCR, only the samples with more than 100 sporangia were scored positive, thus indicating that PCR is less sensitive than LAMP.

## Discussion

The specific detection of pathogens is very important for disease prediction and control. Many rapid molecular detection methods for plant pathogens have been reported. For example, Zeng *et al*.^8^ have developed a nested PCR method to detect the latent infection of wheat leaves by *Blumeria graminis* f. sp. *tritici*. Luminex technology is available to detect more than 20 *Fusarium* species that cause Fusarium head blight of wheat and barley^9^. However, no species-specific detection method for *P. viticola* has been reported previously. Valsesia *et al*.^1^ have developed a quantitative real-time PCR assay to detect the amount of *P. viticola* DNA in leaves treated with potential antagonists and infected with the pathogen. Whether this test can be used for the detection of *P. viticola* under field conditions is unknown because no specificity test of this method on different *Plasmopara* species has been reported. Most rapid and high-throughput detection methods require sensitive and expensive equipment and reagents, such as real-time PCR and Luminex. LAMP is a novel nucleic acid amplification technique that amplifies with high specificity, sensitivity and speed under isothermal conditions. It does not require expensive instrumentation and is therefore more suitable for local on-site detection. Downy mildew is one of the most important grape diseases worldwide. Because of the wide distribution of vineyards in China, regional disease forecasting based on the amount of pathogen and climate is needed. This work was undertaken by Plant Protection Stations in various counties. However, expensive machines, such as real-time PCR equipment, are not currently available in most counties. Therefore, the low-cost LAMP technique is suitable for field application in China. In this study, we developed a highly practical and valid method for the detection of *P. viticola*, which is the causal agent of grape downy mildew. To the best of our knowledge, this is the first report on the application of the LAMP assay to monitor airborne plant pathogens.

On the basis of the ITS sequence, twelve primer sets were designed and tested. No. 12 was selected because of the high specificity and amplification efficiency. With restriction endonuclease profiling and sequencing of the product, we validated the specificity of this primer set for the target region in ITS. In this study, one LoopB primer was developed to accelerate the LAMP reaction described by Nagamine *et al*.^10^. The reaction time was significantly shortened from one hour to half an hour, thereby improving the efficiency of detection.

LAMP is suitable for on-site detection in the field because of its rapidity and robustness, and it does not require elaborate laboratory equipment. For better visibility of the reaction result, a DNA intercalating dye, such as SYBR green^11^,^12^, Picogreen^13^,^14^, or propidium iodide^11^, is added to the solution when the reaction is completed. However, owing to the massive amount of amplification product generated during the reaction, cross contamination with the amplicon from preceding reactions is a major problem that can be circumvented only when the reaction tubes stay unopened after the reaction is finished. Therefore, during the current study, we used a colorimetric assay for LAMP detection by adding HNB as an indirect indicator. Because HNB does not react with the resulting DNA because the color change is dependent on the chelation of Mg^2+^ ions by dNTPs, this indicator can be added before the LAMP reaction^15^. HNB is also superior to calcein, another common pre-added dye^16^, because it produces a more pronounced color change that does not require fluorescence excitation equipment for detection. In the LAMP assay, FIP and BIP primers, which are critical for specificity, hybridize to four binding sites, and the reaction is highly specific. In this study, a set of fungal and oomycete species tested comprised most of the pathogens commonly prevalent on grapes in the field and the closest species within *Plasmopara*. The result indicated that this LAMP assay is highly specific for *P. viticola* and also showed higher sensitivity on both purified DNA and artificially infected leaves than that of conventional PCR. This methodology therefore has great potential for the detection of latent infection on leaves and airborne sporangia.

Because of its high sensitivity, LAMP has been widely used for the early detection of latent infection of clinical^17^,^18^ and agricultural pathogens^19^. To evaluate the possible application of the LAMP assay on infected grape leaves, we tested this method on a collection from 34 counties in 22 different provinces covering all of the main grape-producing areas in China. The crude DNA, including the pathogen, host and other microbes on the surface, was extracted and used as a template. Positive results for all of the leaves from different varieties and geographical regions with apparent symptoms revealed that this method can be used under field conditions. Usually, infection by pathogens does not immediately lead to a disease, and the pathogens can remain latent for a period of time and not produce symptoms until favorable conditions occur. These infected leaves may produce sporangia that serve as an initial inoculum to infect the healthy leaves. However, under unfavorable conditions, such a latent phase can last for a long period of time before symptoms appear. Therefore, an accurate estimation of the levels of latent infection on grape leaves can provide critical information to predict possible disease development in the field. To evaluate the efficiency of this method in the detection of latent infection in *P. viticola*, we compared LAMP and conventional PCR on 72 grape leaves without visible symptoms. The results showed that the detection level of LAMP (65.2%) was nearly threefold higher than that of PCR (22.2%). This result revealed that LAMP is more suitable to estimate the latent infection rate of grape leaves by *P. viticola*. It may be helpful in deciding when the fungicide should be applied and when to reduce the number of fungicide applications to the minimum necessary level. In this study, a rapid and simple method was used to extract crude DNA from the leaf samples within 15 minutes. When this method with the LAMP procedure, the total detecting time was shortened to about one hour. The entire detection process requires no equipment other than a water bath, and this method is highly suitable for the on-site detection of latent infection in the field.

Inoculum levels and weather conditions are usually important factors in disease forecasting^20^. Regarding the monitoring of airborne spores, inoculum-based forecasting schemes have been developed and applied on cucurbit downy mildew (http://cdm.ipmpipe.org/) and soybean rust (http://www.ces.ncsu.edu/depts/pp/soybeanrust/) in the USA. The current understanding of the epidemiology of *P. viticola* suggests that there are two infectious stages in the life cycle. Oospores cause primary infections, and the airborne sporangia released from primary infected leaves are thought to be a secondary inoculum. It has been reported that this secondary inoculum of *P. viticola* is important to the epidemic of grapevine downy mildew^21^. Several disease-forecasting schemes have been developed to aid in control of grape downy mildew in different countries. These schemes are based primarily on weather conditions conducive to oospore formation, infection and sporangia release^22^,^23^,^24^. However, disease would not develop under suitable weather conditions with insufficient inoculum. In this study, we found the disease severity to be strongly associated with airborne sporangia. During this period, there were three rainfall periods recorded, and the number of sporangia in the air increased to a higher level after each rainfall. The first two rainfall periods did not lead to a disease epidemic because there were insufficient sporangia (Fig. 6). This finding revealed the importance of airborne sporangia in addition to the weather in forecasting grape downy mildew epidemics. Traditionally, tape containing air samples on the surface is analyzed under a microscope, and the particles of interest is identified and counted. However, it is difficult to differentiate the sporangia of *P. viticola* from other spores. The LAMP method developed in this study is highly specific to *P. viticola* and simple to perform. Additionally, many tape samples, representing units of time (days or hours), can be tested at one time by one technician; therefore, the LAMP process is much more efficient than counting tape samples one by one under a microscope. LAMP applied to different air samples showed that 66.7% of the samples contained between 20 and 100 sporangia, covering the 21 days before disease outbreak. Before the first rainfall, all of the daily samples were negative, and between the first and second rainfall, only one of six samples was positive; disease did not develop on the leaves thereafter. However, between the second and third rainfall, six of twelve days showed positive results corresponding to the amount of sporangia and the sharply daily increased amount of diseased leaves. This result indicates that the high levels of sporangia in the air and high humidity could result in the outbreak of the disease. On the basis of the results above, considering the estimation of airborne sporangia levels, as determined by LAMP, together with weather conditions will be helpful for decision-making in efforts to control grape downy mildew and to apply chemicals more sparingly, without incurring a risk of yield and quality loss.

## Materials and Methods

### Strains used in this study

The *P. viticola* strains were isolated from diseased grape leaves from Langfang, Hebei Province. A single sporangiophore was picked up and placed on the underside of a healthy grape leaf disc (2 cm in diameter), which was surface sterilized with 70% ethanol and washed with double distilled water. The inoculated leaf disc was placed on water-agar medium in a Petri dish to keep the leaf wet, and the leaves were incubated at 21 °C for 16 h in daylight and 8 h at night and 100% humidity. When white mildew was observed, the sporangias were transferred to other leaf discs for propagation. Sporangia and sporangiophores were collected from the leaf discs and stored in liquid nitrogen.

Another 37 reference strains were also used for the specificity validation of *P. viticola* LAMP and conventional PCR assay. The reference strains belonged to 25 genera, in which there were 8 species within Peronosporales and 9 common grape pathogens. Detailed information, including geographical origin and hosts, is listed in Table 2.

### DNA Preparation

For specificity validation, the pure DNA of *P. viticola* was isolated. Approximately 20 mg of sporangia and sporangiophores were frozen by liquid nitrogen and then were disrupted using a MiniBeadbeater (Biospec, USA). DNA was extracted according to the CTAB protocol described by Möller *et al*.^25^.

A rapid and simple DNA extraction method^26^ was used for DNA preparation from the grape leaf samples. Briefly, a small piece of grape leaf with or without symptoms (approximately 50~100 mg) was transferred to a PCR tube, 50 μL of freshly made buffer A (100 mM NaOH, 2% Tween 20) was added, the mixture was incubated for 10 min at 95 °C, 50 μL of buffer B as added (100 mM Tris-HCl, 2 mM EDTA, pH approximately 2.0), and the samples were mixed at a moderate speed. Finally, 2.5 μL of the solution was used directly as the DNA template for the LAMP and conventional PCR assay.

The DNA of air samples collected by the spore trap were prepared according to the method described by Rogers *et al*.^27^. Sporangia-coated tape sections were placed into 2-mL screw-top tubes, and then 60 μL of MicroLYSIS (Microzone) combined with 0.1 g of 500-μm-diameter glass beads (Biospec, USA) was added and shaken in FastPrep-24 (MP Biomedicals) for 20 s at 4 m s^−1^. The liquid was transferred to PCR tubes and placed in a thermal cycler. The cycling profile was 65 °C for 15 min, 96 °C for 2 min, 65 °C for 4 min, 96 °C for 1 min, 65 °C for 1 min, 96 °C for 30 s, and a hold at 20 °C. Additionally, 2 mg of polyvinylpyrrolidone and 40 μL of TE buffer were added, vortexed and centrifuged to remove polysaccharides. A 60-μL portion of the supernatant was transferred to a new PCR tube, and an ethanol precipitation step was performed. After resuspension of the pellet in 10 μL of H_2_O, 2.5 μL of DNA was used as the template per reaction.

### DNA amplification

With some small modifications, LAMP of *P. viticola* DNA was accomplished as described by Tomita *et al*.^16^. Briefly, 25 μL of the LAMP reaction mixture contained 1.4 μM each of the FIP and BIP primers, 0.2 μM each of the F3 and B3 primers, 0.8 μM of the LB primer, 8 U of *Bst* DNA polymerase (New England Biolabs) and 2.5 μL of 10 × ThermoPol Reaction buffer (including 20 mM MgSO_4_), 0.8 M betaine, 6 mM MgSO_4_, 1.4 mM each of dNTPs, 120 μM hydroxy naphthol blue (HNB) and 2.5 μL of template DNA. The same reaction mixture with 2.5 μL of double distilled water instead of the DNA template was used as the negative control. Reaction tubes were placed in a Veriti 96-Well Thermal Cycler (ABI, USA) operated at a constant temperature of 65 °C for 30 min. The reactions were stopped by heat denaturation of the Bst DNA polymerase for 2 min at 80 °C.

Conventional PCR was performed using the *P. viticola*-specific primers (Pv-cox2F/R). The reaction mixture (20 μL) contained 2.5 μL of DNA template, 0.5 μM of each primer, 10 μL of 2 × PCR Mix (Guangzhou Dongsheng), and 5.5 μL of double-distilled water. The thermal cycler (Veriti 96-Well Thermal Cycler, ABI) was programmed as follows: 95 °C for 5 min, followed by 35 cycles of 95 °C for 30 s, 50 °C for 30 s and 72 °C for 40 s, and finally 72 °C for 7 min. The PCR products were electrophoresed on 1% agarose gels.

### Restriction enzyme digestion and sequence analysis of the LAMP products

To confirm that the LAMP products originated from the correct target fragment, restriction enzyme digestion and sequencing were performed. The LAMP products were digested with the restriction enzymes *Ase* I and *Hinf* I (New England Biolabs) according to the operating instructions. The digested LAMP products were electrophoresed on a 2% agarose gel. Using the F3 and B3 primers, the 228-bp fragment was amplified by PCR and cloned into pMD19-T (Takara, Japan) to create pMD228 for sequencing.

### Specificity of LAMP and the conventional PCR assay

After selection of the optimal primer set, the specificity of LAMP was tested with pure genomic DNA of *P. viticola* and DNA from 37 other reference strains (Table S1). The LAMP and conventional PCR assay were performed and evaluated as described in the previous section.

The specificity of the LAMP reaction was also tested with artificially inoculated grape leaves. Fifty microliters of sporangia suspension (10^4^ sporangia mL^−1^) was coated on the water-agar plate. A small piece of water-agar including one sporangia was cut under the microscope and was transferred to a surface-sterilized healthy leaf. The inoculated leaves were placed on water-agar medium in a Petri dish to keep the leaf wet and were incubated at 21 °C, for 16 h in daylight and 8 h at night and 100% humidity. Three leaves were sampled daily until eight days post inoculation (The symptoms could be observed at 7 dpi). Crude DNA extractions were performed from these samples and were analyzed with LAMP and conventional PCR.

### Sensitivity of LAMP and conventional PCR

The sensitivities of the LAMP assay and conventional PCR were compared using ten-fold serial dilutions of the genomic DNA of *P. viticola* as the template. The initial genomic DNA concentration was 3.3 ng/μL. The LAMP results were visualized by the HNB staining method and were reconfirmed by gel electrophoresis.

### Application of LAMP on *P. viticola*-infected grape leaves

One hundred fifty grape leaf samples comprising 35 varieties were collected from 34 counties in 22 provinces covering all of the main grape-producing areas in China. White mildew on the underside of the leaves was observed in seventy-eight samples (No. 1~78), indicating natural infection by *P. viticola* (Table S1). The other seventy-two samples (No. 79~150) were also collected during the disease period; however, no visible symptoms were observed. To validate the applicable scope of the LAMP assay on naturally infected tissues, LAMP and conventional PCR were both performed on all 150 grape leaf samples, and the results were compared (Table S1). Several leaves with no visible symptoms were placed on water-agar medium and were incubated under the condition mentioned above. Usually, if the leaves were infected by *P. viticola*, white mildew was observed within 7 days. Therefore, after 9 days, leaves with no symptoms were regarded as non-infected samples. One sample from each county was selected as the negative control.

### Application of LAMP on monitoring airborne sporangia in the field

Air samples were taken from a 7-day continuously recording spore sampler (Burkard, UK) operating outdoors according to the standard method reported by Lacey & West^28^. The field air sampling site was at a vineyard in Changli, Hebei Province with an acreage of 2 ha. The air samples were collected for 40 consecutive days during the period from 1/7/2012 to 9/8/2012. During this period, 100 grape plants were selected randomly, and the number of diseased leaves was investigated and recorded daily. Samples from the operational spore traps were counted daily with a microscope and were used for DNA extraction described above. The DNA was identified by both LAMP and conventional PCR.

## Additional Information

**How to cite this article**: Qin, W. *et al*. Development and application of loop-mediated isothermal amplification (LAMP) for detection of *Plasmopara viticola*. *Sci. Rep.* **6**, 28935; doi: 10.1038/srep28935 (2016).

## Supplementary Material

Supplementary Information

## Figures and Tables

**Table 1:** Sequences of LAMP and conventional PCR primers for *P. viticola.*

Primer		Sequence (5′ → 3′)
LAMP	FIP	GAAGCCAACCATACCGCAAATCGGCGACCAATTTATTTGCTGTTG
	BIP	GAATCGGTGAACCGTAGCTATATGTAAGCTGCCACTCTACTTCG
	F3	GTTTGTCTATTGTGGCCAGTC
	B3	CCAAATGGATCGACCCTCG
	LB	GACTATGCTTTCAATCAGTTT
PCR	Pv-cox2F	CAAGATCCAGCAACTCCAGTTATGGA
	Pv-cox2R	ACATTGTCCATAAAAAACACCTTCTC

**Table 2:** Strains used for the specificity validation of LAMP and conventional PCR primers.

Number	Latin name	Host	Source	LAMP	PCR
1	*Plasmopara viticola*	Grape	China	+	+
2	*Plasmopara halstedii*	Sunflower	Germany	−	−
3	*Plasmopara* sp.	Creeper	Germany	−	−
4	*Plasmopara halstedii*	Sunflower	France	−	−
5	*Plasmopara angustiterminalis*	Cocklebur	China	−	−
6	*Peronospora farinosa*	Goosefoot	China	−	−
7	*Phytophthora capsici*	Chili	China	−	−
8	*Phytophthora boehmeriae*	Cotton	China	−	−
9	*Pythium* sp.	Asparagus	China	−	−
10	*Coniella diplodiella*	Grape	China	−	−
11	*Botrytis cinerea*	Grape	China	−	−
12	*Botryosphaeria rhodina*	Grape	China	−	−
13	*Botryosphaeria dothidea*	Grape	China	−	−
14	*Uncinula necator*	Grape	China	−	−
15	*Colletotrichum gloeosporioides*	Grape	China	−	−
16	*Pestalotia mangiferae*	Grape	China	−	−
17	*Guignaridia bidwellii*	Grape	China	−	−
18	*Cryptosporella viticola*	Grape	China	−	−
19	*Alternaria alternata*	Tobacco	China	−	−
20	*Phyricularia grisea*	Rice	China	−	−
21	*Fusarium oxysporum*	Cotton	China	−	−
22	*Fulvia fulva*	Tomato	China	−	−
23	*Exserohilum turcicum*	Maize	China	−	−
24	*Bipolaris maydis*	Maize	China	−	−
25	*Fusarium graminearum*	Wheat	China	−	−
26	*Botryospuaeria berengeriana*	Apple	China	−	−
27	*Botrytis cinerea*	Tomato	China	−	−
28	*Valsa mali*	Apple	China	−	−
29	*Puccinia striiformis*	Wheat	China	−	−
30	*Botryosphaeria ribis*	Poplar	China	−	−
31	*Rhizoctonia solani*	Cotton	China	−	−
32	*Fusarium sp.*	Apple	China	−	−
33	*Blumeria graminis*	Wheat	China	−	−
34	*Ceratobasidium cornigerum*	Wheat	China	−	−
35	*Puccinia triticina*	Wheat	China	−	−
36	*Botrytis cinerea*	Berry	China	−	−
37	*Phaeosphaeria nodorum*	Wheat	China	−	−
38	*Penicillium digitatum*	Orange	China	−	−
